# Three-Tier Plate, Triple Win: Health, Sustainability, and Equity in the Slovenian Nutrition Guidelines 2025

**DOI:** 10.3390/foods15040656

**Published:** 2026-02-11

**Authors:** Nataša Fidler Mis, Boštjan Jakše, Samo Kreft, Ana Vovk, Zlatko Fras

**Affiliations:** 1 Independent Researcher, 1000 Ljubljana, Slovenia; 2 Independent Researcher, 4280 Kranjska Gora, Slovenia; bj7899@student.uni-lj.si; 3 Independent Researcher, 6215 Divača, Slovenia; samo.kreft@gmail.com; 4 Faculty of Arts, University of Maribor, 2000 Maribor, Slovenia; ana.vovk@um.si; 5 Division of Medicine, Centre for Preventive Cardiology, University Medical Centre Ljubljana, Ljubljana, Slovenia and Faculty of Medicine, University of Ljubljana, 1000 Ljubljana, Slovenia; zlatko.fras@kclj.si

**Keywords:** healthy and sustainable diets, EAT–Lancet planetary health diet, food-based dietary guidelines (FBDGs), Slovenian Nutrition Guidelines 2025, plant-based diets, Mediterranean diet, vegetarian diet, whole-food, health equity, environmental sustainability

## Abstract

The prevalence of diet-related noncommunicable diseases (NCDs; e.g., obesity, type 2 diabetes, cardiovascular disease, and certain cancers) is increasing globally, while food systems are also driving climate change and biodiversity loss. Transitioning to predominantly plant-based (“plant-forward”) dietary patterns can improve health and lower environmental impacts. We present the Slovenian Nutrition Guidelines 2025 (SNG2025)—their methodology, development, and core recommendations. Developed as adult food-based dietary guidelines, the SNG2025 are evidence-informed, drawing on the scientific literature, national nutritional data, and expert consensus. We set quantitative daily intake targets by integrating evidence on primary NCD outcomes with environmental metrics (greenhouse gas emissions, land use, and water use), which led to upper limits for animal-based foods. The recommended plant-forward dietary pattern, aligned with the EAT–Lancet planetary health diet, emphasises vegetables, fruits, legumes, whole grains, nuts, seeds, and unsaturated oils; allows low-to-moderate amounts of seafood, poultry, dairy, and eggs; and keeps red and processed meat, free sugars, refined grains, saturated fat, salt, ultra-processed foods (UPFs), and alcohol to a minimum. For the first time, we operationalise health, environmental sustainability, and equity (cultural diversity and accessibility) through a three-tier, plant-forward food plate model (Mediterranean, vegetarian [lacto-ovo], and whole food, plant-based [vegan]). The SNG2025 aim to reduce the risk of NCDs, lower the dietary environmental footprint, and improve fair access to healthy food. They signal a shift from disease management to a prevention-oriented, systems approach that aligns health and ecological goals. With robust implementation, supportive policies, and multisector collaboration, the SNG2025 can strengthen population health, foster more resilient food systems, and advance equity and long-term sustainability.

## 1. Introduction

Dietary patterns are among the most significant and modifiable determinants of both human and planetary health. Globally, suboptimal diet quality is a leading driver of premature morbidity and mortality, fuelling the increasing prevalence of noncommunicable diseases (NCDs), such as cardiovascular disease, type 2 diabetes, obesity, and diet-related cancers [1,2]. In parallel, existing food systems substantially contribute to environmental degradation, including greenhouse gas (GHG) emissions, deforestation, freshwater depletion, and biodiversity loss [3,4]. Addressing these dual challenges requires a profound transformation of dietary guidance and food policy, with a shift towards more plant-based eating patterns [3,5,6].

A ‘plant-based diet’ model is designed to protect both human and planetary health. It outlines flexible intake ranges for food groups, enabling adaptation across regions and cultural contexts while ensuring nutritional adequacy. It refers to a broad spectrum of dietary patterns characterised by reduced quantities of animal-source foods and increased amounts of plant-source foods [7,8]. The EAT–Lancet Commission on Healthy Diets from Sustainable Food Systems proposed a planetary health diet as a flexible reference model designed to safeguard both human and environmental health; it provides adaptable intake ranges by food group to accommodate regional and cultural contexts while maintaining nutritional adequacy [4,9,10]. Reflecting the accumulating evidence, many countries are revising their food-based dietary guidelines (FBDGs) to extend beyond nutrient adequacy and to include sustainability, food system resilience, and equity [11,12].

The World Health Organization (WHO) and Food and Agriculture Organization of the United Nations (FAO) define sustainable healthy diets as those that promote all aspects of health and well-being; that are associated with low environmental pressure and impact; that are accessible, affordable, safe, and equitable; and that are culturally acceptable [13]. This concept aligns closely with the United Nations Sustainable Development Goals (SDGs), particularly SDG 2, which aims to improve the sustainability of food systems [14]. Food production and consumption patterns are major drivers of global environmental change, with food systems accounting for approximately one-third of total GHG emissions [3]. Animal-source foods impose the greatest environmental burden because of their intensive demands for land, water, and energy, as well as indirect impacts such as deforestation and biodiversity loss [4,15]. In contrast, plant-based diets can substantially reduce environmental pressures, particularly within high-income nations such as Slovenia. Legumes, grains, and vegetables, for instance, generate 10–50 times fewer GHG emissions per kilogram than ruminant meat does, and they require considerably less agricultural land [15].

Substituting animal-based foods with plant-based foods could reduce land use by 15–50%, depending on the degree of replacement. While a shift to such diets generally reduces total water use, some plant-based foods, such as fruits, nuts, and pulses, may rely more heavily on irrigation, particularly in water-scarce regions [16]. Thus, a national adaptation of plant-forward models, such as the EAT–Lancet diet, must consider local food cultures, agricultural capacity, nutrient composition, and availability to ensure feasibility and long-term sustainability [4].

Despite the consensus on direction, policy progress remains slow [4] and is hampered by entrenched agricultural subsidies, industry interests, and a biomedical paradigm that emphasises treatment over prevention [17,18]. The commercial determinants of health—aggressive marketing for ultra-processed foods (UPFs), corporate lobbying, and regulatory inertia—further distort food environments, disproportionately affecting low-income and marginalised populations [19,20,21,22]. It is also important to address nutritional equity, recognising gaps in access to plant-forward options in public institutions and workplaces [13].

As recent reviews emphasise, a durable dietary transformation requires not only technical guidance but also political will and regulatory mechanisms to counter commercial interference and align food environments with public health goals [17,22,23]. A 2022 global review of 95 national FBDGs across 100 countries revealed that many still marginalise plant-based diets: fewer than half referenced vegetarian options, and even fewer acknowledged plant-based alternatives to meat or dairy. The Balanced Food Choice Index had a global average score of only 33.6 out of 100; Slovenia scored 55, highlighting systemic biases in dietary recommendations, particularly in countries that are economically dependent on livestock sectors [24]. This gap between scientific evidence and national policy highlights the need for structural reform to align dietary guidance with current knowledge on human and planetary health.

This paper presents the SNG2025, a nutritionally adequate, environmentally sustainable, and culturally adapted plant-forward model that is aligned with the EAT–Lancet planetary health diet [4]. While several recent national dietary guidelines have incorporated sustainability considerations (e.g., Austria [25], Germany [26], the Nordic countries [27,28], and Canada [29]), the SNG2025 is distinctive in formally operationalising health, environmental sustainability, and equity as coexisting normative pillars within a single quantitative framework, implemented through three normatively equivalent plant-forward dietary models. “Plant forward” denotes a predominantly plant-based pattern that allows modest amounts of dairy, eggs, fish, and meat. We describe the methods, evidentiary base, development process, and core recommendations. The SNG2025 aim to reduce the burden of major NCDs (e.g., obesity, type 2 diabetes, cardiovascular disease (CVD), and several cancers) while lowering the dietary environmental footprint [4]. For the first time in Slovenia, these guidelines explicitly integrate health, environmental sustainability, and equity (cultural diversity and accessibility) and apply an inclusion principle [30]—nobody is left out—through three plant-forward plates: Mediterranean, lacto-ovo vegetarian, and whole food, plant-based. Building on two decades of national Mediterranean guidance [31,32], the updated plate is explicitly plant-forward: it contains higher targets for legumes, whole grains, vegetables, fruits, nuts and seeds; tighter caps on red/processed meat and cheese; limits on refined grains and free sugars; using nontropical oils by default; and remaining compatible with modest fish/eggs/dairy consumption. Quantitative daily intake targets and ranges (Table 1 and Table 2) were derived by pairing high-quality evidence on major NCD outcomes with environmental metrics (greenhouse gas emissions, land use, and water use). Table 3 provides concise qualitative guidance. Collectively, the SNG2025 aim to lower NCD risks, reduce the dietary environmental footprint, and reorient national advice towards prevention, sustainability, and equity.

The SNG2025 have undergone a full external scientific review, and all reviewer revisions have been incorporated. The final version of the SNG2025 was formally delivered to the Minister of Health on 8 October 2025, and is currently undergoing governmental approval. Therefore, the guidelines are complete and scientifically finalised but not yet officially adopted or in force.

## 2. Materials and Methods

### 2.1. Context and Policy Integration

The SNG2025 were developed in alignment with national policy priorities and supported by international collaboration. The methodological details and evidence synthesis are described in [33]. The process followed a structured, transparent, multidisciplinary approach that integrated nutritional, health, and environmental criteria. The initiative was launched by the Slovenian Strategic Council for Nutrition (February 2023) and was formally commissioned and overseen by the Ministry of Health. It was subsequently incorporated into the revised National Action Plan for Nutrition and Physical Activity for Health 2015–2025 (September 2023) [34]. In October 2023, a core working group of ten interdisciplinary experts was convened and scientifically led by Prof. Nataša Fidler Mis until external reviews were received (May–June 2024). Thereafter, the coordination of revision and finalisation (July 2024–July 2025) was overseen by Prof. Dr. Zlatko Fras. Hereafter, “we” refers to the core working group (members listed in Supplementary Table S1) [35].

### 2.2. Methodological Framework and Governance

The methodological framework was grounded in the national manual for evidence-based guideline development [36]. A core working group led the scientific work, and an independent review and expert group provided oversight. The process adhered to a structured protocol: (1) the identification and selection of existing high-quality guidelines; (2) adaptation and contextualisation to the Slovenian setting; and (3) the updating of recommendations by using recent evidence on health and environmental outcomes.

Phase 1 comprised a synthesis of existing high-quality systematic reviews, integrating evidence from meta-analyses and global reference frameworks to derive food group recommendations aligned with human and planetary health objectives [33,35]. We then contextualised this evidence through a national situational analysis, considering prevailing dietary intake patterns, the characteristics of the national food system, and the burden of diet-related NCDs in Slovenia.

Operationalisation of dairy recommendations was achieved as follows. Quantitative guidance for milk and dairy products was derived by integrating evidence on nutritional adequacy with environmental impact assessments within the SNG2025 methodological framework. Given the heterogeneous and inconclusive associations between total dairy intake and major health outcomes, together with substantial variation in environmental footprints across dairy products—particularly the markedly greater impacts of cheese compared with milk and yoghurt—the SNG2025 do not define a fixed recommended intake.

Instead, dairy intake was operationalised using a milk-calcium equivalent (MCE) approach, in which milk, dairy products, and calcium-fortified plant-based alternatives were compared on the basis of their calcium content. This enabled the definition of a flexible intake range of 0–500 g/day (milk equivalents). Practical calcium equivalents and substitution examples are provided in Supplementary Table S2 and Supplementary Box S2.

### 2.3. Source Guidelines and Adaptation Approach

The process included the evaluation, selection, adaptation, and updating of existing high-quality guidelines and involved both the core working group and review/expert groups. Three comprehensive, scientifically rigorous guidelines served as the basis for adaptation: the EAT-Lancet Commission report on healthy and sustainable diets [4], the Nordic Nutrition Recommendations 2023 (NNR) [27], and the Canadian FBDGs [29]. The selection criteria included breadth of scope, methodological quality, an explicit treatment of sustainability, and relevance to the Slovenian population. Contextual adaptation drew on the national dietary intake and health status data [35]. Where Slovenian intake patterns or newly published evidence diverged from the source guidance, specific recommendations were revised accordingly [37,38,39,40,41,42].

### 2.4. Evidence Review and Synthesis

We conducted the evidence review independently and supplemented it with systematic searches of PubMed and EMBASE (January 2022–March 2024). We focused on meta-analyses and systematic reviews of prospective cohort studies and randomised controlled trials (RCTs) in adult populations. The primary outcomes were all-cause and cause-specific mortality and incident cardiovascular disease, cancer, incident type 2 diabetes, and bone fractures. When primary outcomes were unavailable, we considered validated intermediate markers (body composition/BMI, blood pressure, blood lipids, fasting glucose/HbA1c, and bone mineral density) to support biological plausibility.

Each included study underwent critical appraisal for methodological quality, funding sources, and potential conflicts of interest (e.g., industry affiliations or consultancy roles). Discrepancies in interpretation and any COI-related concerns were resolved through expert consensus. The resulting synthesis is transparent, methodologically conservative, and contextualised to the Slovenian population, reflecting the most robust evidence available within the review period.

The overall quality and strength of evidence were assessed using a structured, hierarchical approach. Priority was given to systematic reviews and meta-analyses of prospective cohort studies and RCTs evaluating hard clinical endpoints (e.g., all-cause mortality, CVD, type 2 diabetes, and cancer). When evidence on hard endpoints was unavailable or inconsistent, intermediate biomarkers (e.g., blood lipids, blood pressure, glycaemic markers, or body weight) were considered supportive evidence and interpreted cautiously. The strength of the evidence underpinning the recommendations was determined through expert consensus, considering the consistency of the findings across study designs, dose–response relationships, biological plausibility, and relevance to the Slovenian population.

### 2.5. Expert Consultations, Stakeholder Engagement, Review and Revision

We held two expert–stakeholder consultations: (i) an online expert workshop in February 2024 [43], held in collaboration with the WHO Regional Office for Europe, chaired by Vesna Kerstin Petrič, Head of the Office for Cooperation with the WHO [44]. The session opened with welcome remarks by Vesna Kerstin Petrič (Ministry of Health, Slovenia), Melita Vujnović (Head, WHO Country Office, Slovenia), and Kremlin Wickramasinghe (Regional Adviser for Nutrition, Special Initiative on NCDs and Innovation, WHO Regional Office for Europe). (ii) The second consultation was an in-person expert meeting held in May 2024, hosted by the Ministry of Health and the Ministry of Environment, Climate and Energy together with the National Institute of Public Health, chaired by Prof. Dr. Nataša Fidler Mis [33]. The May 2024 expert meeting was opened by the Minister of Health, Dr. Valentina Prevolnik Rupel, Melita Vujnović (WHO Country Office, Slovenia), and Mojca Gabrijelčič (National Institute of Public Health), underscoring the policy relevance and stakeholder engagement. Government representatives played no role in the evidence appraisal, data analysis, or the formulation of final recommendations. In addition, we presented scientific progress and preliminary findings at the 25th Slovenian Forum on Cardiovascular Disease Prevention (March 2024), chaired by Prof. Dr. Zlatko Fras, who made three contributions to the strategic, health, and environmental aspects of the SNG2025 and thus enhanced their transparency and policy relevance [45,46,47].

Following these consultations, five international reviewers and nine national experts (the extended working group) conducted a formal external review (May–June 2024). We collated all the feedback via a structured online platform and revised the SNG2025 from July 2024 to June 2025. In response to the comments of the reviewers and experts, we incorporated 121 new references (sustainability; Mediterranean and planetary dietary patterns; metabolic and mental health; food processing/UPFs; plant-based alternatives and protein quality; and selected disease outcomes—e.g., cancer, dementia—and salt). External reviewers and national experts provided nonbinding advisory input; the core working group retained complete editorial control and responsibility for the final recommendations. To reflect the most recent scientific evidence and policy updates, we added 18 additional references published between April 2024 and June 2025. The final corpus comprises more than 1000 references, ensuring scientific rigour and contemporary relevance.

In parallel, we prepared a plain-language consumer guide derived from the scientific recommendations. Before national release, this guide will undergo a public consultation; feedback will be used to improve clarity and usability without altering the evidence base or final recommendation thresholds.

The entire SNG2025 development process was publicly funded by the Ministry of Environment, Climate and Energy and the Ministry of Health (Slovenia). Cross-ministerial sponsorship facilitated logistical coordination and stakeholder access; the core working group retained complete editorial control and responsibility for all final recommendations [35]. The SNG2025 exemplify a multisectoral, evidence-informed, participatory approach that integrates nutrition science and environmental sustainability, and it provides a framework that other countries may adapt to align dietary guidance with planetary health principles.

### 2.6. Plate Models and Terminology

**Figure 1:**
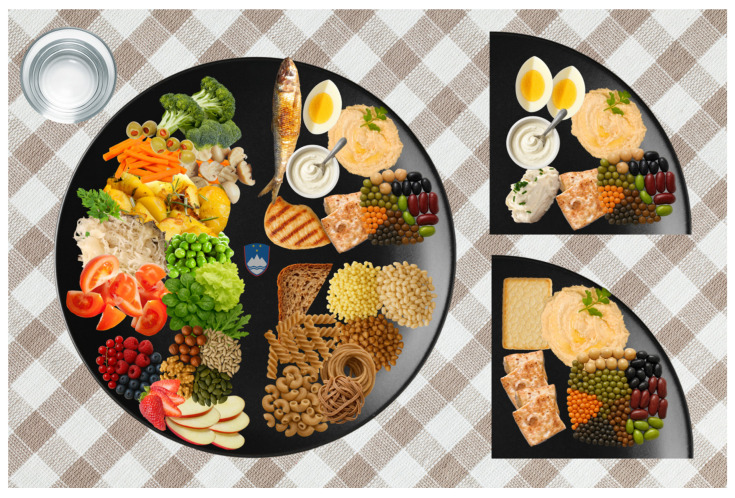
Slovenian Nutrition Guidelines 2025 (SNG2025): Framework and three plant-forward plate models—Mediterranean, lacto-ovo vegetarian, and whole food, plant-based (WFPB).

Throughout this paper, ‘plant forward’ refers to a predominantly plant-based pattern that allows for modest amounts of dairy, eggs, fish, and meat. Vegetarian denotes any vegetarian pattern (lacto-ovo, lacto-, ovo, or pesco-vegetarian); for quantification, Table 1 and Table 2 illustrate the lacto-ovo variant as the reference example. Whole food, plant-based (WFPB) refers to a vegan pattern that emphasises whole or minimally processed plant foods, excludes all animal-source foods, and limits UPFs [8,48]; we use WFPB to distinguish it from generic “vegan” patterns that may include more UPFs. These definitions are applied consistently across the three SNG2025 plate models—Mediterranean, vegetarian, and WFPB—illustrated in Figure 1. The quantitative targets are specified in Table 1 and Table 2, with concise qualitative guidance in Table 3. Vitamin B12 supplementation is required for the WFPB dietary approach and is recommended for vegetarians if the intake of fortified foods is inconsistent (see the table notes). Supplementary Box S1 provides a practical dietary toolkit (sodium and UPFs, meat choices, and cooking methods) aligned with prior Slovenian FBDGs and the new SNG2025. Supplementary Box S2 extends the guidance on potatoes and milk/dairy (including fortified alternatives), and Supplementary Table S2 presents a practical MCE consistent with that of the SNG2025.

## 3. Results

### 3.1. What Are the SNG2025, and Whom Are They for?

The SNG2025 constitute a form of FBDG, offering population-level recommendations structured around food groups rather than isolated nutrients. They represent the first national recommendations in Slovenia to holistically integrate nutritional goals with environmental sustainability and the principle of social equity. They respond to three significant intersecting societal challenges: the increasing prevalence of diet-related NCDs, ecological degradation, and inequitable access to nutritious food. The guidelines are context specific, reflecting national dietary patterns, public health priorities, cultural and culinary heritage, environmental constraints, and the structure of domestic food systems, including Slovenian agricultural production.

The SNG2025 are intended for adults aged 18 and older, including individuals with an elevated risk of NCDs such as cardiovascular disease, type 2 diabetes, obesity, and certain types of cancers. However, they are not intended for individuals with manifest or advanced medical conditions that necessitate individualised medical nutrition therapy.

### 3.2. What Do the SNG2025 Include?

The SNG2025 present a three-part package that integrates human health, environmental sustainability, and equitable access to nutritious food.

#### 3.2.1. Scientific Evidence

Part I: Eating for Health and the Planet. Slovenian Nutrition Guidelines 2025 (SNG2025). This part defines a plant-forward, food-based dietary pattern for adults that is aligned with the EAT–Lancet planetary health diet, and it specifies quantitative daily intake targets and aligns them with the supporting health rationale.

Part II: Environmental Sustainability of the SNG2025. This part provides an in-depth environmental assessment of foods/food groups using harmonised indicators—GHG emissions, land use, water use, and biodiversity impacts—and explains how these metrics inform upper limits and prioritisation across food groups.

#### 3.2.2. Plain-Language Dietary Guidance

Part III: The Balanced Plate: Plain-Language Recommendations. This part translates Parts I–II into practical, culturally relevant messages and visuals. It presents three plant-forward plate models—Mediterranean, vegetarian (lacto-ovo), and WFPB—with everyday examples and tips for shopping and cooking.

### 3.3. Core Dietary Recommendations of the SNG2025

The SNG2025 are intended for adults (≥18 years) and are not designed as child- or adolescent-specific dietary guidelines. The SNG2025 promote a plant-forward pattern aligned with the EAT–Lancet planetary health diet. They set quantitative thresholds using two criteria: (i) health effects, supported by high-quality evidence for major NCD outcomes, and (ii) environmental indicators. Table 1 summarises the daily quantitative targets and ranges; Table 2 operationalises them across the three plate models; and Table 3 provides concise qualitative guidance (beverages, UPFs, salt, alcohol, and cooking methods). Across all three plate models, the dietary pattern is anchored in minimally processed plant foods, restricts UPFs, and recommends alcohol abstinence, as specified in Table 1, Table 2 and Table 3. In addition to health and environmental goals, the guidelines advance inclusion and nutritional justice and, for the first time, explicitly integrate animal welfare considerations. The three plant-forward plates are shown in Figure 1. For beverage standards and further qualitative rules, see Table 3; for practical consumer-oriented guidance, see the Supplementary Materials (Supplementary Table S2; Supplementary Boxes S1 and S2). To enhance the accessibility and practical interpretability of the quantitative targets presented in Table 1 and Table 2, illustrative one-day menu examples for each of the three plant-forward dietary plates are provided in the Supplementary Material (Supplementary Table S3).

For total meat intake, 43 g/day represents a daily equivalent derived from a weekly upper limit of ≤300 g. Meat consumption does not need to be every day; therefore, day-to-day intake may range from 0 to 86 g, provided that the total weekly intake does not exceed 300 g. Importantly, the daily equivalent should not be interpreted as a fixed daily allowance, and 86 g/day × 7 days should therefore not be interpreted as a permitted intake.

Supplementation (outside the plates) is also covered by the SNG2025. Vitamin D is recommended seasonally for all adults (e.g., from September to May in Slovenia). Vitamin B12 is required for the WFPB dietary approach and is recommended for vegetarians if the intake of fortified foods is inconsistent. It is important to ensure ≈250 mg/day of eicosapentaenoic acid (EPA) and docosahexaenoic acid (DHA), on average, via oily fish, fish oil, or microalgae-derived EPA and DHA for the Mediterranean dietary approach or microalgae-derived EPA and DHA for the vegetarian and WFPB dietary patterns (see Table 1 and Table 2 notes).

**Table 1:** Healthy reference diet for the Slovenian Nutrition Guidelines 2025 (SNG2025) at 2500 kcal/day.

Food Group	SNG2025 Daily Intake Guidance per 2500 kcal (g/day Unless Noted) ^a^	Notes
Cereals/grains	≥230 g dry (≈600 g cooked) quality: ≥50% whole grains	≥50% whole grains: • Barley, corn, oats, millet, rye, wheat, (wild) rice, amaranth, buck-wheat, and quinoa • Whole-grain products (contain >50% whole grains) <50% refined grains: • White rice; refined wheat flour/products
Potatoes and other starchy tubers	≤200 g cooked, boiled/baked (not deep fried).	Prefer boiled and/or baked; season with herbs/spices; use only small amounts of oil and iodised salt. Avoid chips, fried potatoes, and potatoes with added butter, lard (crackles), or margarine.
Vegetables	≥300 g	Prioritise variety; include cruciferous vegetables, dark leafy greens, coloured vegetables, and mushrooms; prefer seasonal/local • Dark leafy greens (e.g., rocket and spinach) • Coloured vegetables (e.g., tomato, pepper, and pumpkin) • Mushrooms
Fruits	200 g (100–300 g)	Prefer whole fruit; fruit juice does not count towards the fruit target (see beverages).
Pulses/legumes (dry beans, lentils, and peas) Soy foods (soy, tofu/tempeh, etc.)	≥50 g dry legumes (≈100 g cooked legumes) + ≥25 g dry soy foods (≈70 g cooked soybeans or tofu/tempeh (as soy equivalents))	Include lentils, beans, peas, soy, tofu, tempeh, and edamame.
Dairy or fortified plant-based alternatives ^b,c^	250 (0–500) mL “milk/dairy (“as milk”) or calcium-fortified plant = 1 MCE (~300 mg Ca) ^b,c^	1 MCE (≈300 mg Ca) from any one of: • 250 mL milk/yoghurt • 250 mL calcium-fortified plant drink/soy yogurt ^b^ • ≈50–75 g soft cheese (e.g., Camembert and Brie) ^c^ • ≈27–42 g hard/semihard cheese (e.g., Parmesan ≈ 27 g; cheddar/aged Tolminc ≈ 42 g) ^c^ • Prefer reduced-fat and fermented dairy; consider environmental impact: keep cheese portions modest relative to milk/yoghurt. • Limit products with added sugar/salt. • Plant alternatives should be unsweetened and fortified with Ca, vitamin D, vitamin B12, iodine, and protein; a Ca-fortified soy drink is the most comparable nutritionally.
Meat and processed meat ^d^	Total ≤ 300 g/week (43 g/day; day-to-day 0–86 g) ^d^ Prioritise poultry, reduce red and processed meat	Favour poultry; limit red meat; avoid processed meat. Aim for lower intake overall.
Fish and seafood	200 g/week (0–450 g) ≈29 g/day (0–64 g)	Prefer oily fish rich in EPA/DHA (e.g., sardines and salmon); algal oil is a sustainable nonfish option.
Eggs	≤25 g/day ≈≤3 eggs/week	One egg ≈ 60 g.
Nuts and seeds	≥30 g/day	Prefer unsalted, raw or minimally processed.
Oils and fats from whole foods	≤25 g/day oil and/or whole-food fats (avocado, olives) ^e^ nuts/seeds are separate (30 g/day) Limit/avoid: lard/tallow, butter, cream, tropical fat	Use nontropical plant oils (e.g., olive, rapeseed, canola, sunflower, maize, corn, pumpkinseed, flax, linseed, walnut) and/or whole foods (e.g., 100 g avocado (=20 g oil) + 8–10 pitted olives (=5 g oil)) Limit animal fats (e.g., lard, cracklings, butter, and ghee) and tropical oils/fats (palm and coconut oils/fats).
Herbs and spices	Use regularly to minimise salt/sugar	Build flavour; reduce the need for salt/sugar.
Water and nonalcoholic beverages	≈1500 mL/day	Water and mineral water; unsweetened tea as default. Coffee permitted: ≤400 mg caffeine/day, limit/avoid during pregnancy. Fruit juice occasionally: ≤200 mL/day. Avoid SSBs, especially energy drinks.
Alcohol	0 mL	No safe intake established; abstain.
Ultra-processed foods (UPFs) ^f^	Avoid or minimise	Choose foods low in salt, free sugars, and SFA/TFA; avoid confectionery, refined-flour snacks, processed meats, and SSBs. Limits in the fat, sugars and salt rows.
Overall diet orientation ^f,g,h,i^	<10% E SFA <5% E free sugars ^g^ <0.5% E TFA <5 g salt/day ^h^	<10% E from SFA <5% E from free sugars ^g^ <0.5% E from TFA <5 g/day salt ^h^

^a^ Units/weights. Per 2500 kcal; g/day or mL/day; raw edible amounts unless marked ‘cooked’. ^b^ Fortification. Plant-based alternatives (e.g., soy) should be fortified with calcium and vitamin B12; iodine and vitamin D fortification vary by product/brand (check label). Use plain, unsweetened milk/yoghurt—dairy or plant-based. Consider environmental impact: keep cheese portions modest relative to those of milk/yoghurt. ^c^ Dairy allowance and milk-calcium equivalent (MCE). Dairy is optional in the SNG2025; default 250 g/day within 0–500 g/day, expressed as the MCE. A total of 1 MCE (≈300 mg Ca) from any one of the following: 250 mL of milk, 250 g of yoghurt, 250 mL of calcium-fortified plant drink/soy yoghurt, ~50–75 g of soft cheese (e.g., Camembert and Brie), ~27–42 g of hard/semihard cheese (e.g., Parmesan ≈ 27 g; cheddar/aged Tolminc ≈ 42 g). A total of 0.4 MCE (≈120 mg Ca) from any one of the following (“as milk”): 100 mL of milk, 100 g of yoghurt, 100 mL of calcium-fortified plant drink, 100 g of soy yoghurt, ≈20–30 g of soft cheese, ≈11–17 g of hard/semihard cheese (e.g., Parmesan ≈ 11 g, cheddar/aged Tolminc ≈ 17 g). Units: mL for beverages; g for foods [49]. ^d^ Total meat weekly limit. A total of 43 g/day is the per-day equivalent of ≤300 g/week, day-to-day 0–86 g, weekly upper limit ≤ 300 g; do not interpret 86 g/day × 7 as permitted. ^e^ Oils and fats counting rule. A total of ≤25 g/day from plant oils + whole-food fat sources (avocado, olives, and nuts/seeds (30 g/day) were counted separately. 1 Tbsp oil ≈ 10 g (≈10 mL); ½ small avocado (≈50 g edible) ≈10 g fat; 16–20 pitted olives (≈45–60 g drained) ≈5 g fat. Examples include the following: (i) 2½ Tbsp oil (≈25 g), (ii) 1 Tbsp oil (≈10 g) + ½ small avocado (≈10 g) + 6–10 olives (≈5 g), and (iii) 1½ Tbsp oil (≈15 g) + ½ small avocado (≈10 g). Preference: Choose nontropical plant oils (olive, rapeseed, sunflower, maize/corn, pumpkin seed, flax/linseed, and walnut). Limit animal fats and avoid palm/coconut oil/fat. ^f^ Ultra-processed foods (UPFs) are defined as follows: industrial formulations with cosmetic processing; typically high in salt, free sugars, and SFA/TFA; examples are given in the table; avoid/minimise; see salt/sugars/fats rows for limits; practical tips are in Supplementary Box S1. ^g^ Free sugars. WHO definition (added sugars plus sugars in honey, syrups, fruit juices, and juice concentrates) [50]; target <5% E [51]. ^h^ Salt. The WHO adult limit is < 5 g/day (≈<2000 mg sodium/day) [52]. ^i^ Supplementation: The model assumption is as follows: seasonal vitamin D; B12 mandatory for WFPB; EPA + DHA ≈ 250 mg/day from oily fish or algal oil. Data sources: SNG2025. Abbreviations: DHA, docosahexaenoic acid; %E, percent of total energy; EPA, eicosapentaenoic acid; MCE, milk-calcium equivalent; SFA, saturated fatty acids; SSBs, sugar-sweetened beverages; TFA, trans fatty acids; UPFs, ultra-processed foods.

**Table 2:** Daily intake guidance for the three SNG2025 plates (2500 kcal/day).

Food Groups (per Day)	Plant-Forward Plates ^†^		
	Mediterranean ^i^	Vegetarian ^j^	Whole Food, Plant-Based ^k^
Whole grains and products (e.g., rice, wheat, maize, oats, buckwheat, millet, quinoa, and couscous) Quality: ≥50% whole grains	≥230 g dry; ≥600 g cooked	≥230 g raw; ≥600 g cooked	≥230 g raw; ≥600 g cooked
Potatoes and other starchy tubers (e.g., potatoes)	≤200 g cooked, boiled/baked	≤200 g cooked, boiled/baked	≤200 g cooked, boiled/baked
Vegetables Cruciferous (e.g., broccoli, cabbage, cauliflower, and kale) Dark leafy greens (e.g., rocket and spinach) Coloured vegetables (e.g., tomato, pepper, and pumpkin) Mushrooms	≥300 g	≥300 g	≥400 g
Fruits Berries, grapes, and cherries Apples, plums, and pears Bananas, oranges, and kiwis	200 g (100–300 g)	200 g (100–300 g)	300 g
Pulses/legumes (dry beans, lentils, and chickpeas) Soy foods (soy, tofu/tempeh, etc.)	≥100 g cooked legumes; ≈70 g cooked soybeans or tofu/tempeh (as soy equivalents)	≥100 g cooked legumes; ≈70 g cooked soybeans or tofu/tempeh (as soy equivalents)	≥100 g cooked legumes; ≈70 g cooked soybeans or tofu/tempeh (as soy equivalents)
Dairy or fortified plant-based alternatives ^b,c^	250–500 mL milk/yogurt; or 250–500 mL plant-based drink/yogurt; or 60 g soft cheese or 30 g hard cheese ^b,c^	250–500 mL milk/yogurt; or 250–500 mL plant-based drink/yogurt; or 60 g soft cheese or 30 g hard cheese ^b,c^	0 mL dairy 250–500 mL/day fortified, plant-based drink/yogurt (e.g., soy) ^b^
Meat and meat products Red meat (beef, lamb, and pork) Poultry Processed meat	43 (0–86) g/day total; 14 g red meat; 29 g poultry Total ≤ 300 g/week ^d^	0 g/day	0 g/day
Fish and seafood	29 (0–64 g) g/day	0 g/day	0 g/day
Eggs	12.5 (0–25) g/day	12.5 (0–25) g/day	0 g/day
Nuts and seeds Walnuts, hazelnuts, flax, sesame, and pumpkin seeds	≥30 g/day	≥50 g/day	≥50 g/day
Oils and fats from whole foods Olive oil and rapeseed oil Avocado and olives	≤25 g oil; or 100 g avocado + 8–10 olives; or 20 g oil + 8–10 olives (in addition to nuts and seeds) ^e^	≤25 g oil; or 100 g avocado + 8–10 olives; or 20 g oil + 8–10 olives (in addition to nuts and seeds) ^e^	≤25 g oil; or 100 g avocado + 8–10 olives; or 20 g oil + 8–10 olives (in addition to nuts and seeds) ^e^
Herbs and spices	Use regularly	Use regularly	Use regularly
Water, mineral water, and unsweetened tea ^þ^	≈1500 mL/day	≈1500 mL/day	≈1500 mL/day ^¥^
Supplementation (outside the plate) ^§^			
–Vitamin D	September–May	September–May	September–May
–Vitamin B12			Year-round
–EPA/DHA	From algae, fish, or fish oil	From algae	From algae

Note: All values are daily. Weights refer to raw edible portions unless indicated as cooked. Units: g/day and mL/day unless otherwise specified. ^†^ All three plates are plant-forward and aligned with the EAT–Lancet planetary health diet. ^i^ Mediterranean pattern: high in vegetables, legumes, whole grains, fruits, and nuts; olive oil as the main fat; modest fish/eggs/dairy; and minimal red/processed meat. ^j^ Vegetarian (lacto-ovo): all vegetarian patterns are acceptable; this table illustrates the lacto-ovo variant (includes dairy and eggs; excludes meat and fish). ^k^ Whole food, plant-based (WFPB; vegan): excludes all animal-source foods (meat, fish, dairy, eggs, and honey); emphasises whole/minimally processed foods and minimises ultra-processed foods (UPFs); vitamin B12 supplementation is needed. It uses fortified plant-based alternatives (calcium, vitamin B12, and iodine/vitamin D, as available); microalgae oil can also supply EPA/DHA. ^þ^ Water or mineral water and unsweetened tea as defaults; coffee permitted (≤400 mg caffeine/day); fruit juice occasionally (≤200 mL/day); alcohol abstention recommended. ^¥^ May be lower with a higher intake of water-rich fruits and vegetables. ^§^ Supplementation: Vitamin D: 20 µg/day (800 IU/day) as a reference intake for adults; owing to limited cutaneous synthesis at Slovenia’s latitude, seasonal supplementation of 1000–2000 IU/day is recommended for adults following any of the three dietary patterns, particularly during autumn–spring. Vitamin B12: 4 µg/day as a reference intake for adults; supplementation is required for individuals following the WFPB plate and for vegetarians with inconsistent intake from fortified foods, typically via oral supplements providing higher doses (e.g., 25–100 µg/day). EPA/DHA: ≥250 mg/day, on average: Mediterranean plate via 1–2 servings/week of oily fish or fish-oil/microalgae supplements; vegetarian and WFPB plates should use microalgae-derived EPA/DHA (alfa-linolenic acid conversion alone is insufficient). Supplementation refers to over-the-counter supplements used by adults; individual dosing may be adjusted in clinical settings. Cross-references: Table 1—fortification (note b); dairy allowance, milk-calcium equivalent (MCE) and “as milk” conversions (note c), total meat weekly limit, Mediterranean plate (note d), oils and fats counting rule (note e)). Data sources. See Table 1. Abbreviations: DHA, docosahexaenoic acid; EPA, eicosapentaenoic acid; MCE, milk-calcium equivalent; WFPB, whole food, plant-based.

**Table 3:** Core recommendations/cooking methods of the SNG2025 (qualitative, brief).

Domain	Recommendations
Overall dietary pattern	Prioritise vegetables, fruits, whole grains, legumes, nuts, and seeds. Plant-forward alignment with the EAT–Lancet planetary health diet. Three plant-forward plate models: Mediterranean, lacto-ovo vegetarian, and whole food, plant-based.
Protein	Default to legumes/soy, whole grains and nuts/seeds. Consider health and environmental impact. If included, allow modest dairy, eggs, and fish. Keep cheese portions modest relative to milk/yoghurt. If meat is consumed, prefer poultry, keep red meat minimal, and avoid processed meat; see Table 1 and Table 2 for quantitative limits.
Carbohydrates (staples)	Choose intact/whole-grain staples; keep refined grains low.
Fats and oils	Use nontropical plant oils (e.g., olive and rapeseed/canola) as the default. Limit saturated fats; avoid animal-fat products (lard, cracklings, butter, and ghee) and tropical fats (palm and coconut oil/fat).
Beverages	Water, mineral water and unsweetened tea as default; coffee permitted (<400 mg caffeine/day; limit/avoid during pregnancy). Fruit juice occasionally, ≤200 mL/day. Avoid SSBs, especially energy drinks.
Ultra-processed foods (UPFs)	Limit or avoid UPFs (salty/fatty snacks, confectionery, SSBs, and refined flour products). If chosen, prefer items that are low in saturated/trans fat, free sugars and salt; with minimal/no additives; and higher in fibre/protein.
Free sugars	Limit free sugars (see the WHO definition in Table 1 notes); typical sources are listed under UPFs.
Salt	Reduce added salt, season with herbs/spices, use low-salt preparation.
Alcohol	No safe intake established; abstention recommended.
Cooking methods	Prefer steaming, stewing, pressure cooking and moderate-temperature baking. Deep frying/high-temperature roasting only exceptionally. Potatoes should be consumed boiled/baked, not fried; avoid butter/lard/ghee; use minimal oil; and, if salt is used, choose small amounts of iodised salt.
Food waste	Minimise

Note: Quantitative daily targets and ranges are provided in Table 1 and Table 2 (raw edible portions; g/day and mL/day). Abbreviations: SSBs, sugar-sweetened beverages; UPFs, ultra-processed foods.

#### 3.3.1. Health

The SNG2025 prioritise endpoints with the most substantial evidence: reductions in all-cause and cardiovascular mortality; incident cardiovascular disease, type 2 diabetes, and selected cancers; and favourable changes in validated intermediates (LDL-cholesterol, blood pressure, glycaemic control/HbA1c, body weight, and inflammatory markers). Accordingly, the SNG2025 emphasise legumes, whole grains, vegetables, fruits, nuts/seeds, and nontropical vegetable oils on the basis of meta-analyses and RCTs. The guidelines recommend limiting red and processed meat, free sugars, salt, and UPFs; see Table 3 for alcohol and beverage guidance.

#### 3.3.2. Sustainability

In the SNG2025, environmental indicators (GHG emissions and land and water use) are applied alongside health evidence. This leads to the prioritisation of legumes, whole grains, vegetables, fruits, nuts, and seeds (substantially lower footprints) and tighter upper limits for meat and dairy, which have the highest GHG emissions and land use.

#### 3.3.3. Inclusion, Nutritional Justice, and Animal Welfare

For the first time in Slovenia, the SNG2025 apply an inclusion principle (“nobody is left out”) across three plant-forward plates that span the full spectrum—from plant-rich omnivorous to vegetarian to vegan: Mediterranean; vegetarian (all forms are acceptable—lacto-ovo, lacto, or ovo; Table 2 illustrates the lacto-ovo variant); and WFPB (a vegan pattern that emphasises whole, minimally processed foods and limits UPFs). The Mediterranean plate allows modest fish, eggs, and dairy; the vegetarian plate includes dairy and/or eggs but excludes meat and fish; and the WFPB plate excludes all animal-source foods and requires vitamin B12 supplementation (see Table 2 footnotes). This multipattern design improves cultural adaptability and dietary inclusiveness.

A central innovation is the emphasis on nutritional justice: the SNG2025 acknowledge structural barriers to plant-forward choices in public and workplace food environments (e.g., hospitals, long-term care, and institutional catering) and calls for improved access to appropriate options. For the first time, animal welfare considerations are explicitly integrated alongside health and environmental objectives, contributing to a comprehensive framework for sustainable public health nutrition. Diet quality standards apply to all plates (see Section 3.3 and Table 3).

## 4. Discussion

The SNG2025 provide a plant-forward, evidence-informed framework aligned with the EAT–Lancet planetary health diet [4], and it integrates health, environmental sustainability, and equity. Across three plate models (Mediterranean, lacto-ovo-vegetarian, and WFPB), the guidelines prioritise vegetables, fruits, legumes, whole grains, nuts/seeds, and nontropical oils; set upper limits for red/processed meat, salt, and free sugars; and recommend avoiding alcohol. Key quantitative targets and qualitative standards (UPF limits and beverage guidance) are specified in Table 1, Table 2 and Table 3 and Figure 1.

### 4.1. Alignment and National Adaptations

The SNG2025 are broadly aligned with the EAT–Lancet planetary health diet [4]: a plant-forward orientation; clear limits on red/processed meat; and a preference for legumes, whole grains, nuts/seeds, and low amounts of free sugars and salt. These directions are consistent with the WHO and the FAO guidance on healthy and sustainable diets [13,53], and they parallel recent updates to national food-based dietary guidelines in Europe and North America (e.g., Austria, Denmark, Germany, Spain, Sweden, and Canada ([25,26,27,28,29,54,55])). Within the SNG2025 three-plate framework (Mediterranean, vegetarian, and WFPB), all options are plant-forward and aligned with planetary health principles [4]. Consistent with the EAT–Lancet framework, the SNG2025 also support an entirely plant-based option—excluding meat, dairy, eggs, or fish—via the WFPB plate, with vitamin B12 supplementation and the use of fortified foods as needed. The daily quantitative targets and ranges are listed in Table 1, the three plant-forward plate models are listed in Table 2, and the core recommendations are summarised in Table 3.

Several key national adaptations reflect the Slovenian context. For more than two decades, Slovenia has promoted a Mediterranean dietary pattern—high in vegetables and fruits, with limited animal fats and highly processed foods [31,32]. The SNG2025 do not reprise that earlier plate; they adopt an explicitly plant-forward Mediterranean model, with higher targets for legumes, whole grains, vegetables, fruits, nuts, and seeds, and tighter upper limits for red meat and cheese, alongside a strong recommendation to avoid processed meat, in line with the EAT–Lancet planetary health diet for additional health benefits and a lower environmental footprint [4]. Dairy is treated flexibly through the milk-calcium equivalent approach, explicitly accommodating calcium-fortified plant-based alternatives and traditional fermented dairy products. Guidance on fish is situated towards the lower end of the EAT–Lancet range, while eggs (≤3 per week) align with its midpoint. For fats, the SNG2025 set a daily upper limit of ≤25 g oil equivalents (OE) and counted added oils plus avocado and olives towards the same limit (nuts/seeds are accounted for separately); this places the SNG2025 near the lower bound of the EAT–Lancet oil allocation and reflects a preference for whole/minimally processed fats. Pragmatically, the SNG2025 allow up to 200 g/day of cooked potatoes and other starchy tubers (vs. 0–100 g/day in EAT–Lancet), coupled with preparation methods that avoid the addition of saturated fatty acids (SFAs)/trans fatty acids (TFAs). UPFs and beverage standards are addressed explicitly in the guidance (see Table 3). Culinary herbs and spices are promoted as primary flavouring to help reduce added salt and sugars. These choices reflect dual criteria—high-quality evidence on major NCD outcomes together with environmental indicators (GHG emissions and land and water use)—and consider feasibility, affordability, and equity across public and workplace food environments [4]. Overall, the SNG2025 adopt a whole-diet approach centred on diverse, seasonal, locally sourced plant foods that are whole or minimally processed [56,57,58].

Supplementation is positioned outside the plate models: core nutrient needs should primarily be met through whole foods, and evidence-based supplements are used where appropriate. In Slovenia, vitamin D is recommended seasonally (September–May) for all dietary patterns. Vitamin B12 is required for WFPB and advised for vegetarians when the intake of fortified foods is inconsistent. Ensuring adequate amounts of omega-3 long-chain polyunsaturated fatty acids (EPAs and DHAs) is recommended for WFPB/vegetarian patterns, using oily fish, fish oil, or algal-derived supplements for those avoiding fish. This approach is consistent with regional and national reference values (see Table 2 notes) [59,60].

In the context of emerging evidence, our recommendations align with converging evidence that plant-forward patterns reduce the risk of major NCDs and substantially lower environmental burdens, with the most significant environmental gains achieved by reducing red meat and high-impact dairy and shifting protein sources towards legumes, soy, and nuts/seeds [4,15,61,62]. This interpretation is consistent with contemporary regional guidance that integrates sustainability alongside health [25,26,27,28,29,54,55].

### 4.2. Health and Environmental Co-Benefits

The plant-forward pattern—anchored in minimally processed plant foods, restricted UPFs, and recommended alcohol abstinence—is consistent across the three plates (see Table 1, Table 2 and Table 3). Plant-forward dietary patterns centred on minimally processed plant foods are associated with a lower risk of obesity, CVD, hypertension, and type 2 diabetes and with increased longevity and healthy ageing [48,63,64,65]. Meta-analyses have shown that compared with omnivores, vegetarians have an approximately 15% lower incidence of CVD and a 21% lower incidence of CHD [66]. Plant-forward and Mediterranean-style dietary patterns have also demonstrated favourable effects on lipid profiles, inflammatory biomarkers, and blood pressure [67,68,69,70].

Environmentally, plant-forward diets carry the lowest burdens; the most significant gains come from reducing ruminant red meat and high-impact dairy (e.g., cheese) and shifting protein sources towards legumes, soy, and nuts/seeds [3,4,15,61,62]. An entirely plant-based diet is appropriate if well planned—with vitamin B12 supplementation—and offers the most significant environmental benefit [4,27,54,71]. To operationalise these co-benefits nationally, the SNG2025 add explicit limits on UPFs and standardise beverages—water/mineral water and unsweetened tea are the defaults; coffee is permitted; fruit juice can be consumed on an occasional basis; and alcohol is not recommended (Table 3).

Although plant-forward dietary patterns are consistently associated with lower GHG emissions and land use, the SNG2025 acknowledge heterogeneity in environmental impacts across plant-based foods. Potential trade-offs, such as the higher water footprints of certain plant foods, are addressed by emphasising dietary diversity, seasonal and locally produced foods, and whole or minimally processed plant foods. This pattern-based approach avoids overreliance on a narrow set of resource-intensive foods while maintaining nutritional adequacy and environmental responsibility.

### 4.3. Inclusion, Nutritional Justice, and Implementation

*Inclusion and animal welfare.* The SNG2025 apply an inclusion principle (“nobody is left out”) [4,9,10]. By operationalising three plant-forward plates—Mediterranean, vegetarian (lacto-ovo, for example), and WFPB (a vegan pattern emphasising whole/minimally processed foods and limiting UPFs)—the guidelines accommodate diverse preferences and health needs while maintaining nutritional adequacy (vitamin B12 supplementation is required for WFPB). All three models are anchored in minimally processed plant foods, limit UPFs and free sugars, and recommend abstaining from alcohol. All three patterns have been scientifically validated for their positive effects on human health and environmental sustainability [4,25,26,27,28,29,55,71].

This framing aligns with the FAO/WHO definitions of sustainable healthy diets and recent European guidance, which emphasises accessibility and cultural acceptability [27,53]. It also reflects national risk factor data that justify a precautionary stance on alcohol [41,72,73]. In addition, the SNG2025 integrate animal welfare considerations alongside health and environmental goals, contributing to a comprehensive framework for sustainable public health nutrition. Finally, the guidelines recognise that culturally responsive adaptations improve adherence and help reduce nutrition-related disparities [13,27,30,74].

*Adequacy and equity support.* To ensure adequacy and equity, the SNG2025 emphasise appropriate planning around whole/minimally processed plant foods, routine vitamin B12 supplementation for WFPB (and for vegetarians if fortified foods are inconsistent), and fortified alternatives where relevant (see table notes). Recognising cost and availability constraints, the SNG2025 highlight affordable Slovenian staples—potatoes, buckwheat, beans, cabbage, oats, turnips, onions, garlic, apples, pears, and fermented vegetables (e.g., sauerkraut, pickled turnip), mushrooms, herbs and spices—as culturally familiar, plant-forward choices that meet plate targets and support sustainability.

*Food environments and procurement.* Effective uptake requires supportive food environments. The SNG2025 acknowledge structural barriers to plant-forward choices in public institutions and workplaces (e.g., hospitals, long-term care, and institutional catering) and call for improved availability, affordability, and procurement standards consistent with Table 1, Table 2 and Table 3 [13,27,54]. The action case is reinforced by national intake data—low dietary fibre, suboptimal iron intake/status, variability in vitamin B12 status, and low vitamin D intake [37,39,40,42]—and by climate objectives that favour lower-impact patterns [3,61,62]. The SNG2025 balance cultural relevance with scientific rigour, retaining traditional foods when consistent with health and sustainability goals and supporting dietary shifts where evidence indicates public health and environmental benefits [4].

*Implementation levers and shared goals*. Implementation must embed equity criteria in procurement and catering and improve the availability and affordability of plant-forward options across public, workplace, and retail settings [13]. Health-promoting, sustainable eating is also linked to improved well-being and work performance and may reduce employer and system-level costs [75,76]. The transition towards plant-forward diets will become easier as food environments, affordability, and social norms evolve [4,77]. Coordinated, multilevel strategies—fiscal measures (e.g., taxes and subsidies) that address social and economic barriers, food environment reforms, clear front-of-package labelling, nutrition education, and strengthened social infrastructure—are critical for population-level shifts [21,78,79]. Ultimately, the implementation of the SNG2025 should be anchored in a shared goal: a food system that supports health, environmental sustainability, and equity through sustainable practices, food waste reduction, and high-nutrient, low-carbon offerings [13,54,61,62].

*Policy implications and implementation*. Achieving a population-level impact will require the coordinated translation of the SNG2025 into consistent, culturally resonant messages across healthcare, education, and workplace settings. Clear, evidence-based communication and an alignment of procurement and catering standards can create supportive food environments and improve the availability and affordability of plant-forward options. The engagement of stakeholders whose activities may be affected—including public caterers, retailers, and the livestock and conventional agriculture sectors—is essential. The SNG2025 invite collaboration to cocreate healthier, lower-impact food systems rather than entrench opposition. Monitoring and evaluation should track uptake, equity of access, and health and environmental outcomes to iteratively refine implementation.

*Equity, affordability, and real-world applicability.* From an equity perspective, the three plant-forward dietary patterns recommended in the SNG2025 are comparable in overall cost when they are based on seasonally, locally available, and minimally processed foods and can therefore be economically feasible for low-income households. Differences in food expenditure are driven primarily by food choices within each pattern rather than by the dietary model itself, with animal-source foods generally costing more than legumes and other plant protein sources. Direct cost comparisons across dietary patterns are inherently context dependent, as food prices vary by season, product type, and individual preferences; nevertheless, evidence from multiple settings indicates that whole-food, plant-based dietary patterns are typically among the more affordable options and often cost less than comparable Mediterranean, vegetarian, or omnivorous diets when grounded in minimally processed staple foods [80,81,82].

In practice, the effective implementation of the SNG2025 depends on supportive food environments and coordinated action across policy and practice. Relevant policy instruments include nutrition-based procurement standards for public institutions serving adult populations (e.g., hospitals, long-term care facilities, workplaces), ensuring alignment between dietary guidance and food provision. Monitoring and evaluation should integrate indicators across dietary intake (adherence to plate models), health outcomes (NCD risk factors), and environmental metrics (e.g., GHG emissions). Health professionals, educators, and institutional caterers will play key roles in translating the three-plate model into practice.

### 4.4. Strengths and Limitations

*Strengths.* These include a transparent, multidisciplinary methodology; an explicit integration of health and environmental criteria; and adaptation from high-quality, internationally recognised sources [4,27,29] within a flexible, inclusive three-plate framework (Mediterranean, vegetarian, and WFPB). These features enhance scientific rigour, cultural fit, and usability for policy and practice.

*Limitations.* Some endpoints rely on observational evidence, which is susceptible to residual confounding. Environmental indicators draw on life-cycle assessments that vary by method, geography, and data quality. Real-world uptake/effectiveness requires implementation research and monitoring. Quantitative targets are set for a 2500 kcal baseline and need energy scaling; guidance focuses on adults, and sustainability metrics will evolve as new datasets and methods emerge. Therefore, an ongoing evaluation of population adherence and health outcomes is warranted.

### 4.5. Future Research

*Comparative analyses.*, A quantitative comparison of the SNG2025 with the EAT–Lancet diet [4] and prior Slovenian guidelines [83,84,85], as well as an assessment of the alignment of food intake among Slovenian adults (SI.menu 2017/18) [37,38,39,40,41,42] with the SNG2025, has recently been published [86]. (i) Studies should formulate an analysis of the health and environmental consequences of shifting from current intake to the SNG2025 (e.g., projected changes in dietary risk factors/clinical markers and modelled GHG, land, and water footprints; use comparative risk assessment and LCA). (ii) Studies should compare the SNG2025 quantitatively with the EAT–Lancet planetary health diet [4] and with the most advanced, health- and sustainability-integrated national FBDGs from early adopters in Europe and North America [25,26,27,28,29,54,55].

*Adherence and equity should be monitored.* Uptake should be tracked by individual plate model; diet quality and disparities across socioeconomic groups, regions, and settings (e.g., hospitals and workplaces) should also be tracked.

*The outcomes that should be evaluated.* Studies should prospectively assess changes in dietary risk factors (sodium, red/processed meat, free sugars, UPFs, and alcohol) and clinical markers (blood pressure, BMI, lipids, and HbA1c), as well as the incidence of CVD, type 2 diabetes, and selected cancers. In parallel, environmental indicators (GHG emissions, land use, and water use) should be quantified.

Policy levers should be tested. Studies should conduct real-world evaluations of procurement standards, pricing/tax subsidy measures, reformulation, front-of-package labelling, and food waste reduction, including affordability and cost-effectiveness analyses.

Implementation science should be used. Studies should apply frameworks to measure fidelity, reach, acceptability, scalability, and equity across population subgroups.

The data infrastructure should be strengthened. Studies should link dietary surveys with administrative health and environmental datasets and develop an open indicator dashboard to support continuous evaluation.

## 5. Conclusions

There are three pathways but only one future. The SNG2025 translate the best available evidence into a practical, plant-forward framework that is nutritionally adequate, health-promoting, environmentally sustainable, and inclusive. This framework is operationalised through three plate models—Mediterranean, lacto-ovo vegetarian, and WFPB—offering flexible routes that can be adapted across settings without compromising scientific rigour or cultural relevance.

Implemented at scale, the SNG2025 can lower the risk of major NCDs; reduce the dietary environmental footprint by shifting intake towards legumes, whole grains, vegetables, fruits, and nuts/seeds while limiting the intake of high-impact foods; and advance equity by ensuring appropriate options for everyone, including those choosing vegetarian or WFPB patterns.

To realise these benefits, coordinated action is needed. Efforts should be made to integrate the quantitative targets (Table 1 and Table 2) and qualitative guidance (Table 3) into public procurement and catering standards; align reformulation, pricing/tax subsidy measures, and front-of-package labelling with the guidance; improve the availability and affordability of plant-forward choices (including fortified alternatives and evidence-based supplementation where indicated); embed consistent education across health, education, and workplace settings; and routinely monitor health, equity, and environmental indicators to guide iterative refinement.

The SNG2025 mark a shift from treatment to prevention and from individual responsibility to supportive food environments. With committed, multisector implementation, they can deliver healthier people, a lighter environmental footprint, and a fairer food system, providing a transferable model for countries pursuing aligned health and sustainability goals.

## Data Availability

No new data were created or analysed in this study. Data sharing does not apply to this article.

## Supplementary Materials

The following supporting information can be downloaded at: https://www.mdpi.com/article/10.3390/foods15040656/s1, Supplementary Table S1: Core Working Group (Writing Group) for the SNG2025; Supplementary Table S2: Practical calcium equivalents (MCE); Supplementary Table S3. Illustrative one-day menus aligned with the three SNG2025 plant-forward dietary plates (≈2500 kcal/day). Indicative prices based on major Slovenian retailers; Supplementary Box S1: Practical dietary toolkit—salt, UPFs, and cooking methods; Supplementary Box S2: Practical dietary toolkit—potatoes and milk/dairy (consistent with SNG2025). References [87,88,89,90,91,92,93,94,95,96] has been mentioned in the text.

## Abbreviations

CVD	cardiovascular disease
DHA	docosahexaenoic acid
EPA	eicosapentaenoic acid
EAT–Lancet	EAT–Lancet Commission planetary health diet (reference)
FAO	Food and Agriculture Organization of the United Nations
FBDGs	food-based dietary guidelines
GHG	greenhouse gas
HbA1c	glycated haemoglobin
LCA	life cycle assessment
LDL-C	low-density lipoprotein cholesterol
MCE	milk-calcium equivalent
NCDs	noncommunicable diseases
OE	oil equivalents
RCT(s)	randomised controlled trial(s)
SDGs	sustainable development goals
SFA	saturated fatty acid
SNG2025	Slovenian Nutrition Guidelines 2025
SSB(s)	sugar-sweetened beverage(s)
TFA	trans fatty acid
UPF(s)	ultra-processed food(s)
WFPB	whole food, plant-based
WHO	World Health Organization
