# Supplementary material for: Three-Tier Plate, Triple Win: Health, Sustainability, and Equity in the Slovenian Nutrition Guidelines 2025
Source: Foods. 2026 Feb 11;15(4):656. doi: 10.3390/foods15040656 (PMC12939037; doi:10.3390/foods15040656)
Supplement: Supplementary file 1 [file foods-15-00656-s001.zip › foods-4103847-supplementary.pdf]

Supplementary Table S1. Core Working Group for the Slovenian Nutrition Guidelines 2025 (SNG2025).
Members with affiliation superscripts:

Nataša Fidler Mis, BSc, MSc, PhD[1, *]
Martina Bavec, MSc, PhD[2]
Boštjan Jakše, BSc, PhD[3]
Borut Jug, MD, PhD[4,5]
Samo Kreft, MPharm, PhD[6]
Žiga Malek, BSc, PhD[7,8, ***]
Nina Mikec, MSc, PhD[9]
Nana Turk, BSc, MSc[10]
Ana Vovk, Prof., PhD[11]
Zlatko Fras, MD, PhD, FRCP (Lond), FESC, FACC[5,12, **]

Affiliations:
[1]Independent Researcher, Ljubljana, Slovenia
[2]Faculty of Agriculture and Life Sciences, University of Maribor, Maribor, Slovenia
[3]Independent Researcher, Kranjska Gora, Slovenia
[4]Department of Vascular Disease, University Medical Centre Ljubljana, Ljubljana, Slovenia
[5]Faculty of Medicine, University of Ljubljana, Ljubljana, Slovenia
[6]Independent Researcher, Divača, Slovenia
[7]International Institute for Applied Systems Analysis (IIASA), Laxenburg, Austria
[8]Biotechnical Faculty, University of Ljubljana, Ljubljana, Slovenia
[9]Department of Molecular and Biomedical Sciences, Jožef Stefan Institute, Ljubljana, Slovenia, Independent Researcher, Ljubljana, Slovenia
[10]Central Medical Library, Faculty of Medicine, University of Ljubljana, Ljubljana, Slovenia
[11]Faculty of Arts, University of Maribor, Maribor, Slovenia
[12]Division of Medicine, Centre for Preventive Cardiology, University Medical Centre Ljubljana, Ljubljana, Slovenia

Footnotes:
[*]Lead author during the first half of SNG2025 development
[**]Lead author during the second half of SNG2025 development
[***]Lead author of Part II (sustainability) of SNG2025 development

Supplementary Table S2. Practical calcium equivalents (MCE).

Food	Ca (mg/100 g or mL)	1 MCE[1] Amount providing 300 mg Ca	0.4 MCE[2] Amount providing 120 mg Ca
Milk	120	250 mL	100 mL
Yogurt	120	250 g	100 g
Fortified plant-based drink	120	250 mL	100 mL
Fortified plant-based yogurt	120	250 g	100 g
Brie (soft)	400	75 g	30 g
Camembert (soft)	615	50 g	20 g
Cheddar (semihard)	721	42 g	17 g
Aged Tolminc (semihard)	721	42 g	17 g
Parmesan (hard)	1109	27 g	11 g

Note. Milk-calcium equivalent (MCE). [1] 1 MCE (≈ 300 mg Ca) is provided by any one of the following: 250 mL of milk, 250 g of yoghurt, 250 mL of calcium-fortified plant drink, 250 g of soy yoghurt, ≈ 50–75 g of soft cheese (e.g., Camembert and Brie), and ≈ 27–42 g of hard/semihard cheese (e.g., Parmesan ≈27 g, cheddar/aged Tolminc ≈ 42 g). [2] 0.4 MCE (≈120 mg Ca) is provided by any one of the following: 100 mL of milk, 100 g of yoghurt, 100 mL of calcium-fortified plant drink, 100 g of soy yoghurt, ≈ 20–30 g of soft cheese (e.g., Camembert and Brie), and ≈ 11–17 g of hard/semihard cheese (e.g., Parmesan ≈11 g, cheddar/aged Tolminc ≈17 g) [1].

Supplementary Table S3. Illustrative one-day menus aligned with the three SNG2025 plant-forward dietary plates (≈2500 kcal/day). Indicative prices based on major Slovenian retailers.

**Meal** [a, b]	**Plant-forward plates**		
	Mediterranean plates	*Vegetarian*	*Whole food, plant-based plates*
Breakfast	• Whole-grain bread (80 g, 0.35 €) with black/green olives (10 pieces, 30 g, 0.30 €), tomato (100 g, 0.30 €)	• Whole-grain bread (80 g, 0.35 €) with homemade jam (15 g, 0.07 €) (no added sugar)	• Whole-grain bread (50 g, 0.20 €) with avocado (50 g, 0.20 €), hummus (50 g, 0.20 €), tofu (100 g, 0.20 €), tomato (50 g, 0.15 €), arugula/spinach (50 g, 0.10 €), red onion (20 g, 0.03 €)
	• Greek yogurt (250 g, 0.80 €) with oatmeal cereals (50 g, 0.15 €), mixed berries (200 g, 1.50 €), flaxseeds (15 g, 0,10 €), unhulled sesame seeds (15 g, 0,10 €), pitted, unsweetened dates (2-3 medium, 30 g, 0,40 €)	• Greek yogurt (250 g, 0.80 €) with seeds and fruits as in Mediterranean plate	• Soy yogurt (250 g, 0.90 €) with seeds and fruits as in Mediterranean plate
	• Beverages: water (200 mL), green tea (200 mL), or black coffee (unsweetened)	• Beverages as in Mediterranean plate	• Beverages as in Mediterranean plate
Lunch	• Grilled salmon (120 g, 2.40 €) with quinoa/rice (150 g cooked, 0.18 €)	• Baked butternut squash (200 g, 0.40 €) with chickpeas (100 g cooked, 0.15 €), (homemade) tahini sauce (15 g, 0.18 €), and roasted pumpkin seeds (15 g, 0.22 €)	• Tempeh/soy tofu curry (100 g tempeh/soy tofu, 0.20 €) with carrots (100 g, 0.12 €), broccoli (100 g, 0.50 €), and coconut milk (50 mL, 0.07 €), with brown rice (200 g cooked, 0.24 €)
	• Mixed salad (600 g) with seasonal vegetables (e.g., cooked beans (100 g; 0.30 €), potato (100 g; 0.10 €), tomato (50 g; 0.25 €), cucumber (50 g; 0.20 €), cabbage (50 g; 0.10€), corn (50 g; 0.30 €), lettuce (50 g; 0.20 €), radishes (50 g; 0.25 €), carrots (50 g; 0.20 €), pumpkin seeds (30 g; 0.40 €)) dressed with olive oil (5 g; 0.05 €), balsamic vinegar or lemon juice (5 mL; 0.05 €) and iodised salt<br>• Whole-grain bread (2 slice, 80 g, 0.35 €)	• Mixed salad (600 g) as in Mediterranean plate<br>• Brown rice (100 g cooked, 0.12 €) with scrambled egg (1 medium, 60 g, 0.35 €), sautéed spinach (50 g, 0.10 €), tomato (50 g, 0.15 €), red onion (40 g, 0.06 €), cabbage (50 g, 0.07 €), avocado (30 g, 0.12 €), and walnuts (30 g, 0.60 €)	• Mixed salad (600 g) as in Mediterranean plate
	• Fruits: 1 apple (150 g; 0.30 €), 1 banana (100 g; 0.20 €), 1 orange (100 g; 0.25 €)	• Fruits: as in Mediterranean plate	• Fruits: as in Mediterranean plate
	• Beverages: water (200 mL), green tea (200 mL), or black coffee (unsweetened)	• Beverages as in Mediterranean plate	• Beverages as in Mediterranean plate
Dinner	• Chickpea stew (150 g cooked chickpeas (0.22 €), Brazil nut (1 piece), red onions (40 g, 0.04 €), garlic (10 g, 0.12 €), spinach (50 g, 0.10 €), and tomato (50 g, 0.15 €)) served	• Tofu stir-fry (150 g tofu, 0.30 €) with mixed vegetables (broccoli, cabbage, red onions) (200 g, 0.40 €) and quinoa/brown	• Chickpea stew as in Mediterranean plate<br>• Vegan burger (1 patty, 100 g, made from beans, oatmeal cereal, quinoa, red beet, chia seeds, 0.20 €)

	with roasted potatoes (150 g, 0.15 €), soy tempeh/soy tofu (100 g, 0.20 €), and olives (10 pieces, 30 g, 0.30 €)<br>• Steamed broccoli (200 g) with a drizzle of black olive oil (5 g) and garlic<br>• Fruits: 1 apple (150 g; 0.30 €), 1 banana (100 g; 0.20 €), 1 orange (100 g; 0.25 €)	rice (100 g cooked, 0.12 €)<br>• Whole-grain pita (1 piece, 80 g) stuffed with spinach (50 g) and cottage cheese (50 g)<br>• Fruits: as in Mediterranean plate	with whole-grain bread (1 piece, 60 g, 0.22 €) and arugula (40 g, 0.20 €), herbs/spices, and iodised salt, 1 tablespoon (5 g) dried seaweed (nori or wakame)<br>• Fruits: as in Mediterranean plate
Throughout the day	• Water and unsweetened herbal and nonherbal tea (1.5 L)<br>• Optional: 2–3 cups of black coffee without sugar	Same as in Mediterranean plate	Same as in Mediterranean plate

Note. [a] Menu quantities are illustrative and designed for an average healthy adult, aligned with Slovenian national reference values based on Central European (D-A-CH) recommendations [2,3], and verified using the national Open Platform for Clinical Nutrition (OPEN) [1,4]. The menus are not prescriptive but serve to demonstrate alignment with the quantitative targets presented in Tables 1 and 2. [b] Supplementation, where needed, is addressed in the main SNG2025 recommendations and is not expanded on in these illustrative examples. [c] Ingredient costs are illustrative and were calculated on 19 January 2026 using typical retail prices in Slovenia. Prices vary by season, brand, retail setting, and product quality; therefore, the examples are intended to illustrate relative affordability across dietary patterns rather than provide a universal cost comparison. In this scenario, the summed ingredient costs per day were €12.11 for the Mediterranean menu, €12.33 for the vegetarian menu, and €9.36 for the whole-food plant-based menu.

Supplementary Box S1. Practical dietary toolkit: salt, UPFs, and cooking methods (consistent with the SNG2025 and prior Slovenian FBDGs).

Daily salt target

- Keep total salt <5 g/day (≈ <2000 mg sodium/day).
- If any salt is added, use iodised salt.

High-risk UPF categories to minimise

- Minimise UPFs—they are major carriers of "hidden salt" and are often high in free sugars and SFAs/TFAs.
- Limit high-salt/sugar/TFA condiments (processed meats, soy sauce, stock cubes/bouillon, ready spice mixes, marinades, savoury snacks, instant soups/sauces, margarines/shortenings, sweet bakery/confectionery, and SSBs).

Practical swaps:

- Processed meats → legumes/tofu/nuts;
- Refined snacks → nuts/fruit/whole-grain crackers;
- Flavoured yoghurts → plain + fruit;
- Sweetened drinks → water/mineral water/unsweetened fruit or herbal tea.

"Label rules" (sodium):

- "Hidden sodium" additives: sodium chloride, sodium bicarbonate, sodium phosphates, sodium nitrite/nitrate, monosodium glutamate (MSG), baking powders, brines, and marinades.
- Label check: choose the lowest amount of salt per 100 g available. Rule of thumb: ≤0.3 g/100 g = low; ≥1.5 g/100 g = high.

Cooking

- Cook with basic ingredients, favour minimally processed foods, and opt for home cooking.
- Flavour without salt: herbs, spices, garlic/onion, citrus, vinegar, tomato, and mushrooms.
- Minimal added fat should be used; deep-fried/fast foods should be avoided; and high-temperature oil, overcooking and charring should be avoided.

Supplementary Box S2. Practical dietary toolkit: potatoes and milk/dairy (consistent with the SNG2025).

Potatoes and Other Tubers

- Potatoes are essential staples in Slovenia and provide potassium and fibre when prepared in a healthy fashion. They can be included daily, up to 200 g (cooked), preferably boiled or baked. During preparation, saturated or trans fats (e.g., butter, lard, and margarine) should be avoided, and only small amounts of plant oil and iodised salt should be used. Chips and fried potatoes should be limited [5].
- From an environmental perspective, potatoes and other starchy tubers have a low greenhouse gas footprint and modest land and water requirements, making them essential components of a sustainable, plant-forward diet [6–8].

Milk, Dairy, and Fortified Alternatives

- The consumption of milk and dairy products should be determined individually on the basis of overall nutrient intake. A reasonable daily intake may include 250 g (0–500 g) of milk or dairy products or calcium-fortified plant drinks that provide an equivalent amount of calcium as milk [9]. Milk and dairy products are also major dietary sources of SFAs (followed by oils and meat products) [10], and they can also be sources of added sugar and salt [1]. Reduced-fat and fermented dairy are preferred. In contrast, products with added sugar and/or salt should be limited [10].
- Calcium-fortified plant-based drinks and yoghurts (without added sugar) can be considered alternatives to milk and yoghurt [11,12]. Their nutritional quality varies with the ingredients (cereals, legumes, nuts, and blends) and production methods; therefore, fortification with calcium, protein, vitamin D, vitamin B12, and iodine is essential to achieve equivalence. Unsweetened, calcium-fortified soy drink is recognised as the most suitable milk alternative by SNG2025 because its protein content is comparable to that of milk. Other plant-based drinks marketed as "milk alternatives" are not nutritionally equivalent unless they are fortified. Individuals who avoid milk and dairy products should ensure adequate replacement of calcium, vitamin B12, and protein through fortified products or supplements to prevent long-term deficiencies [13–15].
- Although milk generally has lower environmental impacts than many other animal-source foods do, cheese and similar products carry substantially higher burdens for greenhouse gas emissions, land use, water use, and eutrophication [9].

References:

1. Institut Jožef Stefan - Computer Systems Department Computer web-based software: the Open Platform for Clinical Nutrition (OPEN) Available online: http://opkp.si/en_GB/cms/introduction (accessed on Feb 7, 2024).
2. German Nutrition Society, Austrian Nutrition Society, Society for Nutrition Research, S.N.A. *Ergaenzlieferung D-A-CH Referenzwerte für die Nährstoffzufuhr (Reference Values for Nutrient Intake)*; 4th ed.; Frankfurt am Main, 2020;
3. National Institute of Public Health of Slovenia Reference values for energy intake and nutrient intake Available online: https://www.nijz.si/sites/www.nijz.si/files/uploaded/referencne_vrednosti_2020_3_2.pdf (accessed on Feb 7, 2024).
4. Korošec, M.; Golob, T.; Bertoncelj, J.; Stibilj, V.; Seljak, B.K. The Slovenian food composition database. *Food Chem.* **2013**, *140*, 495–499.
5. Borch, D.; Juul-Hindsgaul, N.; Veller, M.; Astrup, A.; Jaskolowski, J.; Raben, A. Potatoes and risk of obesity, type 2 diabetes, and cardiovascular disease in apparently healthy adults: a systematic review of clinical intervention and observational studies. *Am. J. Clin. Nutr.* **2016**, *104*, 489–498.
6. Ivanovich, C.C.; Sun, T.; Gordon, D.R.; Ocko, I.B. Future warming from global food consumption. *Nat. Clim. Chang.* **2023**, *13*, 297–302.
7. Poore, J.; Nemecek, T. Reducing food's environmental impacts through producers and consumers. *Science (80-.).* **2018**, *360*, 987–992.
8. Petersson, T.; Secondi, L.; Magnani, A.; Antonelli, M.; Dembska, K.; Valentini, R.; Varotto, A.; Castaldi, S. SU-EATABLE LIFE: a comprehensive database of carbon and water footprints of food commodities Available

online: https://figshare.com/articles/dataset/SU-EATABLE_LIFE_a_comprehensive_database_of_carbon_and_water_footprints_of_food_commodities/13271111/2 (accessed on Feb 1, 2024).
9. Willett, W.; Rockström, J.; Loken, B.; Springmann, M.; Lang, T.; Vermeulen, S.; Garnett, T.; Tilman, D.; DeClerck, F.; Wood, A.; et al. Food in the Anthropocene: the EAT–Lancet Commission on healthy diets from sustainable food systems. *Lancet* **2019**, *393*, 447–492.
10. Eilander, A.; Harika, R.K.; Zock, P.L. Intake and sources of dietary fatty acids in Europe: Are current population intakes of fats aligned with dietary recommendations? *Eur. J. Lipid Sci. Technol.* **2015**, *117*, 1370.
11. Medici, E.; Craig, W.J.; Rowland, I. A Comprehensive Analysis of the Nutritional Composition of Plant-Based Drinks and Yogurt Alternatives in Europe. *Nutrients* **2023**, *15*, 3415.
12. Ramsing, R.; Santo, R.; Kim, B.F.; Altema-Johnson, D.; Wooden, A.; Chang, K.B.; Semba, R.D.; Love, D.C. Dairy and Plant-Based Milks: Implications for Nutrition and Planetary Health. *Curr. Environ. Heal. Reports* **2023**, *10*, 291.
13. Johnson, A.J.; Stevenson, J.; Pettit, J.; Jasthi, B.; Byhre, T.; Harnack, L. Assessing the Nutrient Content of Plant-Based Milk Alternative Products Available in the United States. *J. Acad. Nutr. Diet.* **2024**, S2212-2672(24)00269–7.
14. Brusati, M.; Baroni, L.; Rizzo, G.; Giampieri, F.; Battino, M. Plant-Based Milk Alternatives in Child Nutrition. *Foods* **2023**, *12*, 1544.
15. Walther, B.; Guggisberg, D.; Badertscher, R.; Egger, L.; Portmann, R.; Dubois, S.; Haldimann, M.; Kopf-Bolanz, K.; Rhyn, P.; Zoller, O.; et al. Comparison of nutritional composition between plant-based drinks and cow's milk. *Front. Nutr.* **2022**, *9*, 988707.
